# The effects of aerobic exercise on eGFR, blood pressure and VO_2_peak in patients with chronic kidney disease stages 3-4: A systematic review and meta-analysis

**DOI:** 10.1371/journal.pone.0203662

**Published:** 2018-09-11

**Authors:** Karsten Vanden Wyngaert, Amaryllis H. Van Craenenbroeck, Wim Van Biesen, Annemieke Dhondt, Anouk Tanghe, Ans Van Ginckel, Bert Celie, Patrick Calders

**Affiliations:** 1 Department of Rehabilitation Sciences and Physiotherapy, Faculty of Medicine and Health Sciences, Ghent University, Ghent, Belgium; 2 Department of Nephrology, Antwerp University Hospital, Antwerp, Belgium; 3 Laboratory of Experimental Medicine and Paediatrics, University of Antwerp, Antwerp, Belgium; 4 Department of Internal Medicine, Renal Division, Ghent University Hospital, Ghent, Belgium; University of Mississippi Medical Center, UNITED STATES

## Abstract

**Background:**

CKD is associated with several comorbidities, cardiovascular disease being the most significant. Aerobic training has a beneficial effect on cardiovascular health in healthy and some well-defined non-healthy populations. However, the effect of aerobic training on glomerular filtration rate in patients with CKD stages 3–4 is unclear.

**Objective:**

To review the effects of aerobic exercise training on kidney and cardiovascular function in patients with chronic kidney disease (CKD) stages 3–4.

**Methods:**

A random-effects meta-analysis was performed to analyse published randomized controlled trials through February 2018 on the effect of aerobic training on estimated glomerular filtration rate, blood pressure and exercise tolerance in patients with CKD stages 3–4. Web of Science, PubMed and Embase databases were searched for eligible studies.

**Results:**

11 randomized controlled trials were selected including 362 participants in total. Favourable effects were observed on estimated glomerular filtration rate (+2.16 ml/min per 1.73m^2^; [0.18; 4.13]) and exercise tolerance (+2.39 ml/kg/min; [0.99; 3.79]) following an on average 35-week aerobic training program when compared to standard care. No difference in change in blood pressure was found.

**Conclusions:**

There is a small beneficial effect of aerobic training on estimated glomerular filtration rate and exercise tolerance, but not on blood pressure, in patients with CKD stages 3–4. However, data are limited and pooled findings were rated as of low to moderate quality.

## Introduction

Patients with chronic kidney disease (CKD) have a high risk to develop cardiovascular (CV) disease, for which exercise training is known to be a successful preventive strategy [1]. Also, the probability for death (with CV disease as leading cause) in patients with CKD stages 3–4 is higher than the probability to progress to end-stage renal disease [2–4]. Therefore alone already it is useful to stress the utility of exercise training. In addition, most patients suffer of a severely impaired exercise tolerance and performance, both in themselves predictors of survival [5]. Underlying mechanisms include renal anaemia, malnutrition, vascular dysfunction and/or polyneuropathy [6–8]. Consequently, the majority of patients with CKD do not meet the European Guidelines for physical activity of 150 min/week of moderate intensity (class I, level of recommendation A) [9]. This creates a vicious circle of worsened exercise intolerance and physical inactivity further enhancing increased CV risks [10]. Exercise tolerance plays a pivotal role in reaching recommended physical activity levels, which in turn is essential in the preservation of functional movement, general well-being and the management of CV risk factors (e.g. body weight control) in patients without CKD. Exercise training improves exercise tolerance and allows individuals to increase their physical activity levels. Some evidence suggests a similar beneficial impact in patients with CKD [11]. Primarily patients with CKD stages 3 or worse [4], i.e. when CV risk starts to increase, could potentially benefit from aerobic exercise training [12].

Despite the absence of a consensus on which frequency, intensity, type and time of exercise is of most benefit, regular exercise increases peak oxygen consumption (VO_2_peak), improves blood pressure control, CV risk profile, mental health and health-related Quality of Life in the entire range of CKD patients [10, 11]. Aerobic exercise, as opposed to anaerobic training, is assumed to induce systemic changes, possibly affecting determinants of glomerular filtration rate. However, there is still a knowledge gap concerning the effect of aerobic exercise on estimated glomerular filtration rate. Therefore, an updated meta-analysis can present new insights on the effects of exercise training with respect to the most recent Cochrane review [11]. In this meta-analysis, we investigated the effect of aerobic exercise in patients with CKD stages 3–4 through a meta-analysis. The following research questions will be answered: What is the effect of aerobic exercise on (1) estimated glomerular filtration rate (eGFR) and (2) blood pressure and VO_2_peak compared to standard care in patients with CKD stages 3–4?

## Materials & methods

### Data sources and selection criteria

This systematic search and meta-analysis was performed according to the Preferred Reporting Items for Systematic Reviews and Meta-Analyses (PRISMA) guidelines [13] and checklist (S1 Checklist). The protocol of this meta-analysis was registered retrospectively on PROSPERO (registration number: CRD42018103223). Only randomized controlled trails (RCTs) were included. Following inclusion criteria needed to be fulfilled: (1) adult (>18 years) human subjects, (2) mean baseline eGFR of the population between 59 and 15 ml/min per 1.73m^2^ (CKD stage 3 and 4), (3) aerobic exercise includes the intervention method, (4) intervention encompassed at least 3 months of training with at least 2 sessions per week and (5) studies had to be written in Dutch, English, German or French. Non-randomized controlled and observational trials, reviews, meta-analyses, short communication articles, letters to the editor and study protocols were excluded. No publication date or other restrictions were imposed. Studies were searched describing the effect of aerobic exercise compared to standard care on eGFR, blood pressure or VO_2_peak in patients with CKD stages 3–4. Three electronic databases were consulted and searched for eligible papers until February 2018 (i.e. Web of Science, PubMed and Embase) with a supplementary search on clinicaltrials.gov to identify potential missing trials and to retrieve protocols. Reference lists of included studies were screened for additional studies. MeSH-terms or keywords used in the search strategy (S1 Table) included: ‘Chronic Renal Insufficiency’, ‘Chronic Kidney Disease’, ‘Chronic Renal Failure’, ‘Exercise’, ‘Circuit-Based Exercise’, ‘Rehabilitation’, ‘Aerobic Exercise Training’, ‘Aerobic Exercise’, ‘Exercise Training’, ‘Glomerular Filtration Rate’, ‘GFR’, ‘Kidney Function’, ‘Renal Function’, ‘Heart Rate’, ‘Blood Pressure’, ‘Oxygen Consumption’, ‘VO_2_peak’ and ‘Aerobic Capacity’. Search strategy, full selection process, screening on title, abstract, full text (Kappa: 0.97), data extraction and quality assessment were developed and executed in duplicate by 2 independent assessors (VWK and TA, PhD students Rehabilitation Sciences and Physiotherapy, under the supervision of CP, PhD). Discrepancies were discussed and in case of no unanimous conclusion referred to CP, who had the final decision.

### Data extraction and quality assessment

Qualitative (Table 1) and quantitative data were extracted from the included articles. Corresponding authors were addressed to provide missing or tailored data based on our eligibility criteria (e.g. excluding patients with CKD stage 2). Data for between-groups analyses was extracted from the results of patient and control group analyses. Body mass index (BMI) was post hoc included as secondary outcome. Methodological quality of studies was evaluated according the checklist for assessing risk of bias provided by the Cochrane Collaboration [14]. Quality of evidence and strength of recommendations were assessed by the Grading of Recommendations, Assessment, Development and Evaluations (GRADE) method [15].

**Table 1:** Table of evidence and characteristics of included studies.

Study	Purpose and primary outcome	Exercise group		Control group	Intervention	Time frame	Kidney parameters	Cardiovascular parameters	Main results
Aoike et al (2015)	Home-based aerobic exercise provides physical and clinical benefits in OP with CKD; Primary outcome ^a^:VO_2_peak	9♂, 5♀; BMI 31.7±4.5; GFR 28.4±11.6; Age 55.9±7.7	10♂, 5♀; BMI 30.7±4.1; GFR 25.3±13.4; Age 54.3±8.7		Aerobic exercise training: 3 sessions/week: 5min warming up/cooling down; 30min walking at heart rate at VT 1 obtained in CPET and spirometry; +10min each 4 weeks; At start 3 supervised sessions	12 Weeks	GFR	Heart rate; Blood pressure; VO_2_	Increased heart rate at VT; Decreased heart rate in rest; Decreased resting systolic and diastolic blood pressure; Improvement GFR; Improvement peak- and VT-VO_2_
Baria et al (2014)	Impact of aerobic exercise on visceral fat in CKD obese patients; Primary outcome ^a^: Reduction visceral fat	Centre based; 10 ♂; BMI 30.8±5.1; GFR 25.8±8.8; Age 52.1±11.4—Home based; 8 ♂; BMI 30.9±3.9; GFR 29.4±11.2; Age 50.8±7.7	9 ♂; BMI 29.6±1.9; GFR 27.7±15.0; Age 53.4±9.6		Aerobic exercise training: Centre based, 3 sessions/week: 5min warming up/cooling down; 30min walking at heart rate at VT 1 obtained in CPET and spirometry; +10min each 4 weeks—Home based, 3 sessions/week: Exercise with uniform instructions as centre-based group; +10min each 4 weeks during one supervised session	12 Weeks	GFR	Blood pressure; VO_2_	Improvement GFR and blood pressure in centre-based group; No improvement GFR and blood pressure in home-based group; No improvement VO_2_peak
Greenwood et al (2015)	Effects of exercise on GFR and cardiovascular function; Primary outcome ^a^: eGFR	6♂, 2♀; BMI 27.4±3.52; GFR 36.6±10.1; Age 53.8±13.5	9♂, 1♀; BMI 28.4±4.24; GFR 46.5±20.6; Age 53.8±13		Aerobic and resistance exercise training: 2 supervised sessions/week (20min); 1 unsupervised session/week (40min); 5min warming up / cooling down; 80% heart rate reserve cycling with maximal heart rate obtained in CPET; 80%1RM resistance training	52 Weeks	GFR	Blood pressure; Heart rate; VO_2_	Improvement mean rate of change in GFR compared the 12 months pre-intervention period; No between-group change GFR; No change resting blood pressure and heart rate; Improvement relative VO_2_peak; No change absolute VO_2_peak
Headley et al (2012)	Exercise improves heart rate recovery and VO_2_peak in predialysis kidney patients; Primary outcome ^b^: eGFR	10 patients; BMI 32.7±7.2; GFR 33.2±20.1; Age 57.5±11.5	11 patients BMI 34.2±5.7 GFR 48.5±23.4; Age 57.5±11		Aerobic and resistance exercise training: 3 supervised sessions/week:; 5min warming up/cooling down; 45min at HR at 50–60% VO_2_peak obtained in CPET and spirometry; Treadmill, cycle, elliptical machines, stairmasters; Week 24 to 48: Resistance training to avoid boredom	48 Weeks	GFR; Proteinuria	blood pressure; Heart rate; VO_2_	No change in GFR, creatinine clearance and proteinuria; No change in resting ambulatory blood pressure; Decreased resting ambulatory heart rate; Improvement VO_2_peak
Headley et al (2014)	Effects short-term aerobic exercise on vascular function in CKD; Primary outcome ^a^: Pulse wave velocity	25 patients; BMI 34.9±8.0; GFR 47.0±12.0; Age 58.0±8.0	21 patients; BMI 36.5±8.9; GFR 48.3±12.7; Age 57.1±9.0		Aerobic exercise training: 3 supervised sessions/week: 5min warming up/cooling down; 45min at HR at 50–60% VO_2_peak obtained in CPET and spirometry: Treadmill, cycle, elliptical machines, stairmasters	16 Weeks	/	Blood pressure	No change blood pressure
Howden et al (2013)	Effect of exercise and lifestyle intervention on cardiovascular function in CKD; Primary outcome ^a^: VO_2_peak	36 patients; BMI 32.5±6.8; GFR 38.4±8.8; Age 60.2±9.7;	36 patients; BMI 33.0±8.0; GFR 39.4±8.9; Age 62.0±8.4		Aerobic and resistance exercise training: 3 supervised sessions/week for 8 weeks; 5min warming up/cooling down; 30min treadmill, cycling and rowing at 11–13 on RPE-scale; 3 unsupervised session/week:; 150min exercise/week at 11–13 on RPE-scale	52 Weeks	GFR	Blood pressure; VO_2_	No change in GFR and blood pressure; Improvement VO_2_peak; More patients reached age-predicted exercise tolerance
Leehey et al (2009)	Effect of aerobic exercise in obese patients with CKD; Primary outcome ^b^: /	7♂ ; Diabetes Mellitus type 2 ; GFR 23.3±12.0 ; Age 66	4♂ ; Diabetes Mellitus type 2 ; GFR 30.4±13.2 ; Age 66		Aerobic exercise training: 3 supervised sessions/week for 6 weeks; 5min warming up/cooling down; Week 1–3: 6min 25–44%, 18min 45–59% and 6min 60–84% of VO_2_peak; Week 4–6: 6min 25–44%, 22min 45–59% and 12min 60–84% of VO_2_peak; 3 unsupervised sessions/week: Increase step count by 10%/week; Intensity based on CPET & spirometry	24 Weeks	GFR; Proteinuria	Blood pressure; Heart rate; VO_2_	Decreased resting systolic blood pressure, no effect after follow-up; No change GFR and heart rate; Decreased proteinuria; Improvement VO_2_peak; Tendency improvement VO_2_ at isotime (at the exact same workload)
Leehey et al (2016)	Exercise in obese patients with CKD; Primary outcome ^a^: Urine protein to creatinine ratio	14♂; BMI 36.2±4.8; GFR 41.5±18.8; Age 65.4±8.7	18♂; BMI 37.4±4.2; GFR 38.9±20.3; Age 66.6±7.5		Aerobic and resistance exercise training: 3 supervised sessions/week for 12 week; 60min interval training on treadmill, elliptical machine and cycle at 25–84% HR at VO_2_peak obtained in CPET; 30min resistance training: 3 (60min) or 6 (30min) unsupervised sessions/week with similar instructions	52 Weeks	GFR; Proteinuria	Blood pressure; VO_2_	No change GFR and proteinuria; No change resting systolic blood pressure and VO_2_peak
Miele et al (2016)	Lipoprotein pattern and response to moderate aerobic exercise in CKD; Primary outcome ^b^: HDL-pattern	25 patients; BMI 34.9±8.0; GFR 47±12	21 patients; BMI 36.5±8.9; GFR 48.3±12.7		Aerobic exercise training: 3 supervised sessions/week; 15min to 55min aerobic exercise 50–60% VO_2_peak obtained on CPET and spirometry	16 Weeks	GFR	VO_2_	No change GFR; Improvement VO_2_peak
Mustata et al (2011)	Effects of exercise on arterial stiffness in predialysis CKD; Primary outcome ^b^:/	7♂, 3♀; BMI 27.5; GFR 27±?; Age 64±4.5	6♂, 4♀; BMI 29; GFR 28±?; Age 72.5±3.5		Aerobic exercise training: 5 sessions/week:; 2 supervised + 3 unsupervised sessions; 20min at 40–60% VO_2_peak obtained in CPET:; Treadmill, cycle, elliptical trainer; Duration +10% each week to 60min	52 Weeks	GFR	Blood pressure; VO_2_	No change in GFR and blood pressure; Improvement VO_2_peak
Van Craenenbroeck et al (2015)	Effect of 12 weeks home-based exercise training on endothelial function in CKD 3–4; Primary outcome ^a^: Flow-mediated dilation	11♂, 8♀; BMI 28.3±6.2; GFR 37.5±13.2; Age 51.5±11.8	11♂, 10♀; BMI 28.3±5.8; GFR 39.6±12.9; Age 54.7±14		Aerobic exercise training: 4 sessions/day; 10min cycling/session at 90% heart rate at VT obtained at CPET and spirometry; 3 supervised sessions in week 1–2; 1 supervised session in week 3–4	12 Weeks	GFR	Blood pressure; VO_2_	No change in GFR and blood pressure; Improvement peak- and VT-VO_2_; More P reaching age-predicted VO_2_peak

♀ = Female Patients; ♂ = Male Patients; BMI: Body Mass Index; CG = Control Group; CKD = Chronic Kidney Disease; GFR = Glomerular Filtration Rate in ml/min/1,73m^2^; METs = Metabolic Equivalents; Min = Minutes; RM = 1 Repetition Maximum; RPE = Borgs’ Rating of Perceived Exertion Scale; VT = at Ventilatory Treshold (ml/kg/min)

^a^ Power calculation was performed at ≥80%

^b^ No power calculation was performed

### Statistical analysis for meta-analysis

The ‘GRADEpro Guideline Development Tool’ and ‘Review Manager 5.3.5’ from the Cochrane Collaboration were used for quality assessment of evidence and to calculate effect estimates for combinations of single effects from included studies and to subsequently perform subgroup analyses respectively. Treatment effects were calculated as mean differences in outcomes. If eGFR was calculated using another equation than the CKD-EPI or Modified Diet in Renal Disease formula (MDRD), the CKD-EPI formula was applied on mean and standard deviation values of the population [16]. The Dersimonian-Laird inverse variance weighted random effects method was used to pool study findings and heterogeneity was calculated (I^2^, <25% = no heterogeneity and ≥75% = high heterogeneity) [17]. Sensitivity analysis was planned and subgroup analyses were performed on changes in eGFR to explore sources of heterogeneity and subgroup variables included: intervention type (i.e. aerobic exercise vs. resistance and aerobic exercise) and duration, age and BMI. Age and BMI were categorised based on cut-off points: 60 years old and 32 kg/m^2^ respectively. Subgroup breakdown for intervention duration was based on duration more or less than 6 months. Results were considered significant at 95% study confidence interval. Forest plots were used to present findings of the meta-analysis.

## Results

### Search results

**Fig 1:**
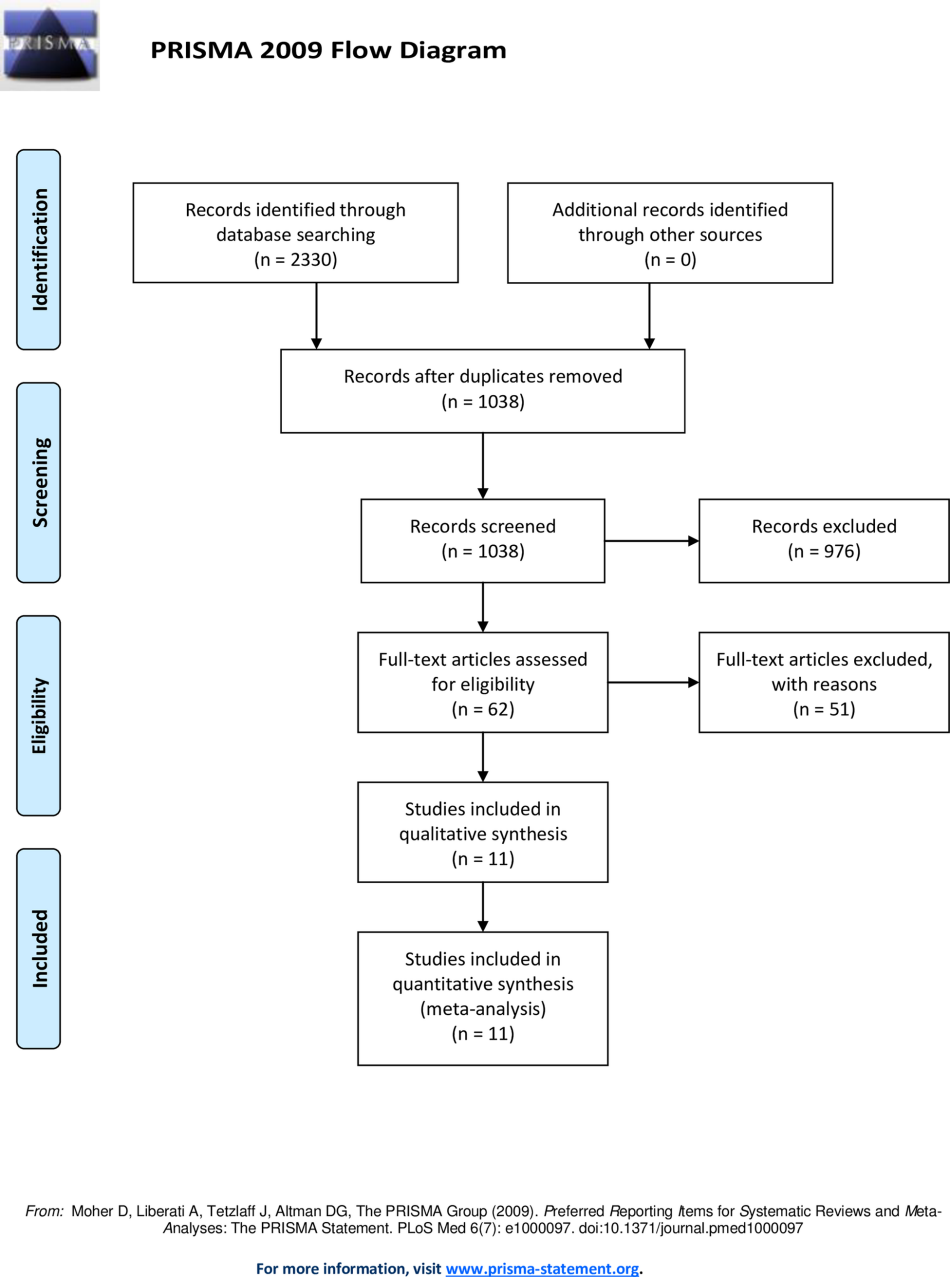
PRISMA flow diagram for the systematic review and meta-analysis.

A total of 1038 studies were identified through our database search. After removing duplicates and two screening phases (title/abstract and full text), 11 studies were withheld based on the in- and exclusion criteria (Fig 1).

### Study characteristics

Depending on the study and following the ‘Kidney Disease Outcomes Quality Initiative’ (KDOQI) guidelines [18], participants presented with stages 2–4 [19–21], 3–4 [22–26] or stage 3 [1, 27, 28] CKD. Four studies had a therapy duration of 6 months or more [1, 19, 24, 25] and aerobic exercise modalities included: (1) walking [20, 22, 23, 25], (2) cycling [24, 26] or (3) use of various training-devices like treadmill, cycle ergometer, elliptical machines, stairmasters or rowing machines [1, 19, 21, 25, 27, 28]. Resistance training was included as intervention in four studies [1, 19, 21, 24]. Training intensities were monitored based on: (1) heart rate at 90% [26] or 100% [22, 23] heart rate at ventilatory threshold, (2) percentage of intensity at VO_2_peak (i.e. 50–60% [19, 27, 28], 40–60% [25] and 25–84% [20, 21]), (3) heart rate at 80% of heart rate reserve [24] and (4) Borg Rating of Perceived Exertion scale 11–13 [1].

### Quality assessment

**Fig 2:**
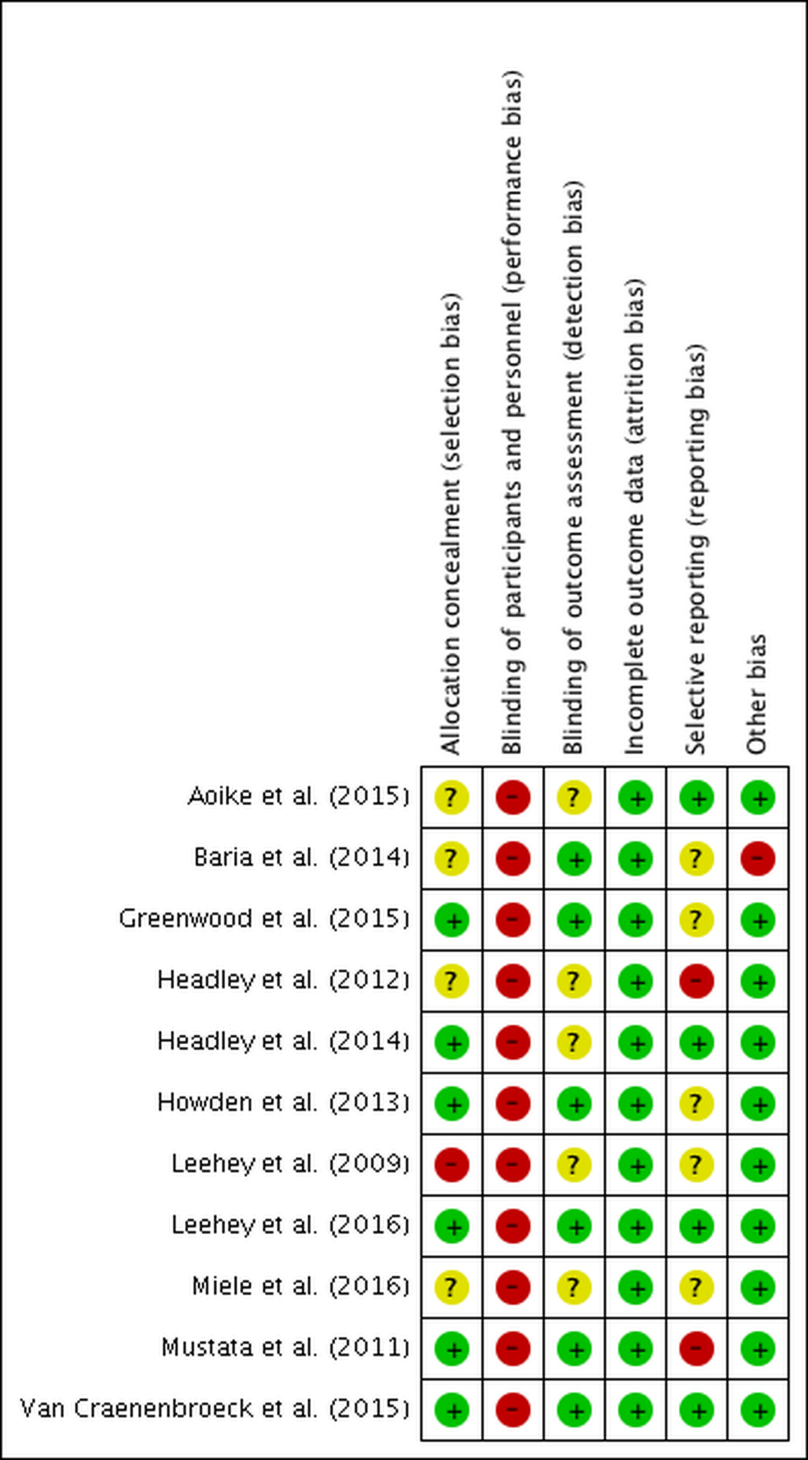
Risk of bias.

Risk for selection and detection bias was unclear in four (36%) [19, 22, 23, 28] and five (45%) [19, 20, 22, 27, 28] RCTs respectively. Only Leehey et al was scored as high risk for selection bias due to allocation concealment [20]. Because blinding of participants is an insurmountable obstacle in exercise intervention RCTs, high risk of detection bias was granted to all RCTs. Five studies scored unclear risk for reporting bias based on lacking registration on clinical trial registries [1, 20, 23, 24, 28]. Attrition and the other sources of bias performed better compared to the aforementioned bias criteria with a low risk in more than 75% of the studies (Fig 2). Quality of evidence for the eGFR and BMI analyses were rated as of moderate strength deriving from detection bias (Table 2). Findings of blood pressure and VO_2_peak were valued of poorer quality due to high heterogeneity (i.e. I^2^≥75%) and blood pressure outcome was rated of high risk for publication bias based on asymmetrical funnel plots (S1–S5 Fig).

**Table 2:** Quality assessment of outcome (GRADE).

Certainty Assessment						№ of Patients		Effect	Certainty	Importance (/9)
№ of Studies	Study Design	Risk of Bias	Inconsistency	Indirectness or Imprecision	Other Considerations	Exercise Group	Control Group	Absolute (95% CI)		
Glomerular Filtration Rate										
10	RCT	+ ^a^	+ ^b^	-	Strong Association	151	154	2.16 ml/kg per 1.73m^2^; [0.18; 4.13]	⨁⨁⨁◯ (Moderate)	Crucial (7)
Systolic Blood Pressure										
8	RCT	+ ^a^	++ ^c^	-	Publication Bias Strongly Suspected ^d^	133	136	1.22 mmHg; [-4.45; 6.90]	⨁◯◯◯ (Very Low)	Not Important (2)
Diastolic Blood Pressure										
7	RCT	+ ^a^	++ ^c^	-	Publication Bias Strongly Suspected ^d^	119	118	0.06 mmHg; [-3.22; 3.34]	⨁◯◯◯ (Very Low)	Not Important (2)
Exercise Tolerance										
11	RCT	+ ^a^	++ ^c^	-	Strong Association	161	164	2.39 ml/kg per min; [0.99; 3.79]	⨁⨁◯◯ (Low)	Important (6)
Body Mass Index										
8	RCT	+ ^a^	+ ^b^	-	Strong Association	144	150	-0.73 kg/m^2^; [-1.38; -0.09]	⨁⨁⨁◯ (Moderate)	Important (6)

**-** = Not Serious; + = Serious; ++ = Very Serious; CI = Confidence Interval; RCT = Randomized Controlled Trials

^a^ High risk for detection bias

^b^ 25%<I^2^<75%

^c^ I^2^≥75%

^d^ High risk for publication bias based on funnel plots

### Training-induced effects

#### Estimated glomerular filtration rate

**Fig 3:**
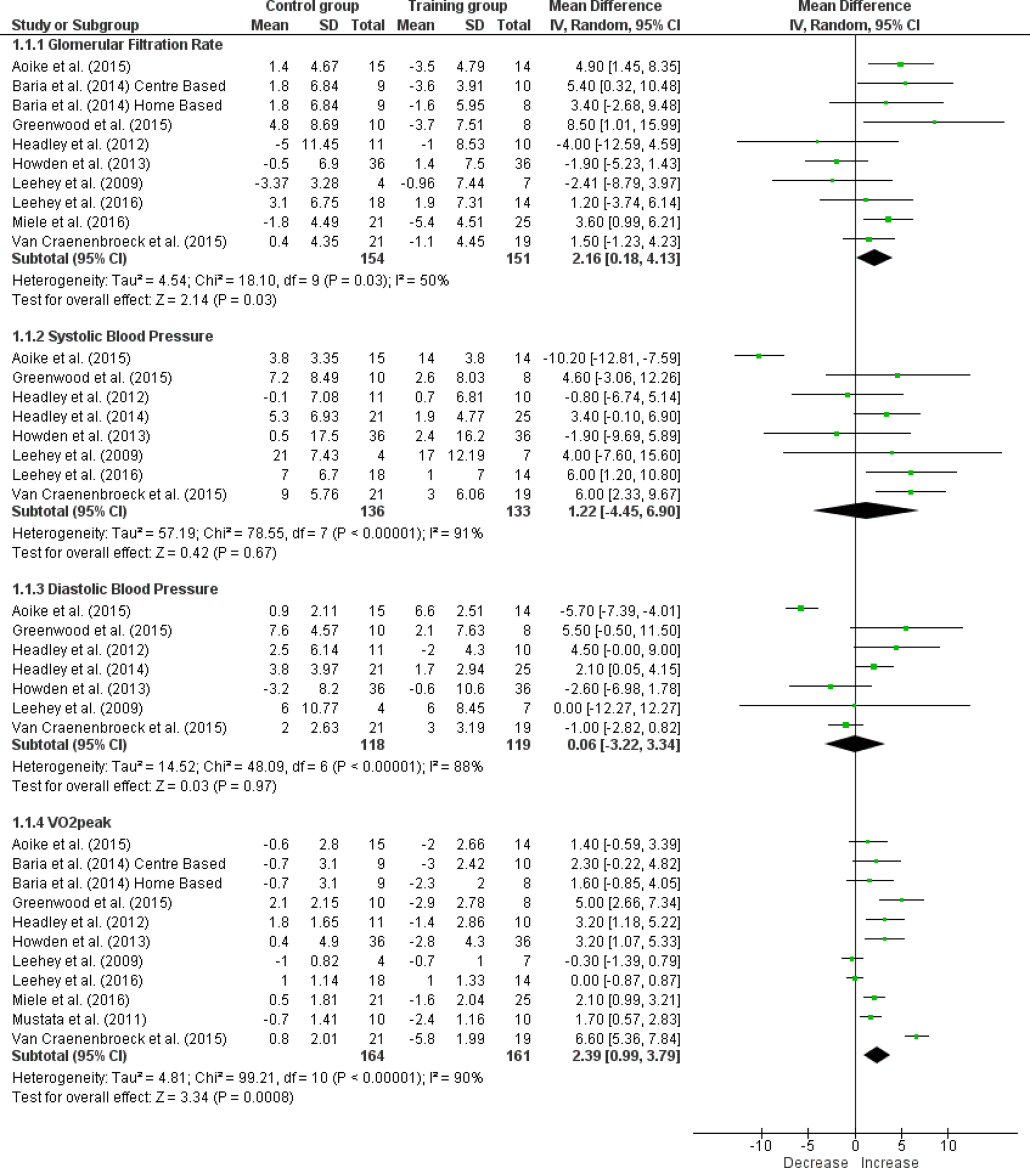
Forest plots between-groups analysis. (1) glomerular filtration rate (ml/min per 1.73m^2^), (2) systolic blood pressure (mmHg), (3) diastolic blood pressure (mmHg) and (4) VO_2_peak (ml/kg/min).

**Fig 4:**
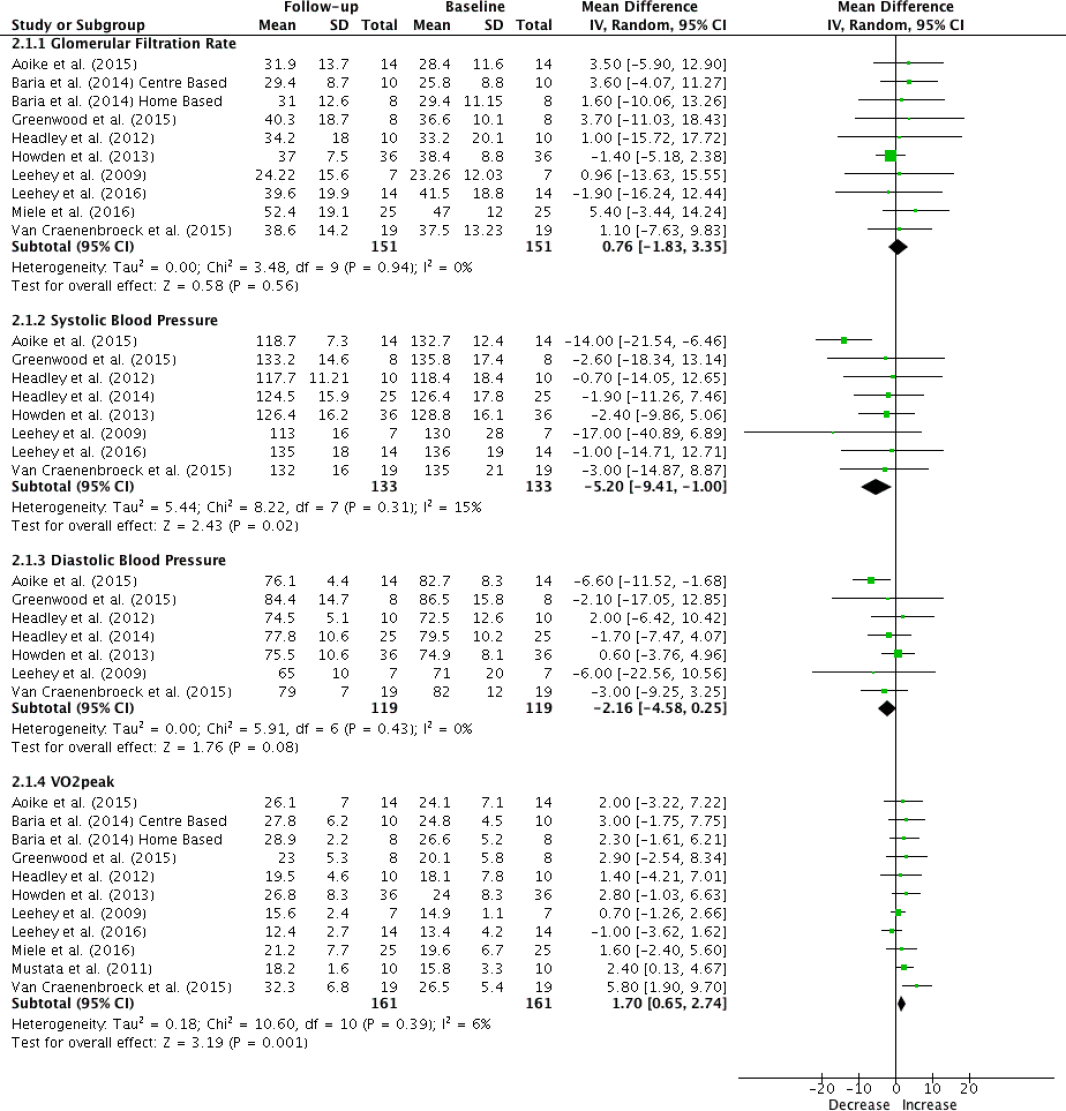
Forest plots within-group analysis (training group). (1) glomerular filtration rate (ml/min per 1.73m^2^), (2) systolic blood pressure (mmHg), (3) diastolic blood pressure (mmHg) and (4) VO_2_peak (ml/kg/min).

Ten studies assessed eGFR as endpoint kidney function parameter [1, 19–26, 28]. eGFR was obtained using the CKD-EPI creatinine equation in three studies [22–24, 29] and the MDRD formula in seven studies [1, 16, 19–21, 25, 26, 28]. Nine RCTs compared aerobic exercise with standard care in, respectively, 151 and 154 participants over a mean period of 35 weeks [1, 19–24, 26, 28]. One study was excluded based on incomplete reporting of data [25]. Duration of intervention varied between 12 weeks [22, 23, 26] and one year [1, 21, 24]. A calculated mean difference shows an improvement in eGFR of 2.16 ml/min per 1.73m^2^ [0.18; 4.13] (I^2^ = 50%) in participants allocated to aerobic exercise compared to standard care (Fig 3). No significant within-group changes in eGFR were established (Fig 4 and S6 Fig).

#### Blood pressure

Ten studies described the effect of aerobic exercise on blood pressure in patients with CKD stages 3–4. For systolic and diastolic blood pressure, respectively, eight and seven studies were included in the meta-analysis covering a total of 133 and 119 patients receiving aerobic exercise compared to 136 and 118 control subjects [1, 19–22, 24, 26, 27]. Two and three studies were excluded for systolic [23, 25] and diastolic [21, 23, 25] blood pressure analysis based on incomplete reporting of data respectively.

Blood pressure measurements took place in a sitting position after at least 5 minutes rest and the average of 2 readings when pressures differed for less than 6 mmHg [19, 27] or of 3 readings [19, 22, 24, 26, 30] was used. Protocol was not clearly stated in three studies [1, 20, 21].

Aerobic exercise training of a 32-week duration on average showed no effect on blood pressure in the between-groups analysis (Fig 3). However, mean decreases in systolic blood pressure of 5.20 mmHg [1.00; 9.4] (I^2^ = 15%) in patients assigned to aerobic exercise (Fig 4) and of 4.92 mmHg [1.10; 8.75] (I^2^ = 7%) in patients receiving standard care were obtained (S6 Fig).

#### Exercise tolerance

Ten studies examined exercise tolerance, as quantified by VO_2_peak in CKD stages 3–4. These studies compared 161 participants receiving aerobic exercise with 164 control subjects [1, 19–26, 28]. Similarly, over an intervention period of on average 32 weeks, meta-analysis of the VO_2_peak showed a within-group increase of 1.70 ml/kg/min [0.65; 2.74] (I^2^ = 6%) amongst patients following aerobic exercise training (Fig 4). When compared to standard care, aerobic training improved VO_2_peak by 2.39 ml/kg/min [0.99; 3.79] (I^2^ = 90%) (Fig 3).

#### Body mass index

Eight studies were included in the analysis of BMI covering 144 patients receiving on average 32 weeks aerobic exercise training compared to 150 control subjects [1, 19, 21–24, 26, 27]. A decrease of 0.73 kg/m^2^ [-1.38; -0.09] (I^2^ = 54%) was found in favour of the exercise group (S7 Fig).

#### Subgroup analyses

Analysis performed on changes in eGFR outcome with studies categorised mean age <60 or ≥60 years old showed a difference disfavouring the elderly (p = 0.004; I^2^ = 88%) (S8 Fig). Also a subgroup difference with mean BMI 32 kg/m^2^ as cut-off point was noted (p<0.001; I^2^ = 92.2%); in favour of subjects scoring <32 kg/m^2^ (S9 Fig). Leehey et al was excluded in the latter analysis due to a nutritional intervention in the control group [21]. No subgroup differences were found when studies were divided based on intervention duration and type (S10 and S11 Figs).

## Discussion

The purpose of this meta-analysis was to evaluate the effects of aerobic exercise training in patients with CKD stages 3–4 on outcomes of cardiovascular and kidney function when compared to standard care. We found that aerobic exercise improved eGFR (2.16 ml/min per 1.73m^2^), VO_2_peak (2.39 ml/kg/min) and decreased BMI (-0.73 kg/m^2^) when compared to standard care. In line with the most recent Cochrane review [11], aerobic exercise may thus be beneficial for the CV risk profile. We did not find differences in blood pressure following an aerobic training program compared to standard care. Hence, our study supports European guidelines on physical exercise are also applicable in patients with CKD stages 3–4 [9].

Even though only two studies used exercise tolerance as primary outcome, it is not really surprising to find an increase in VO_2_peak after an aerobic exercise intervention in patients with CKD stages 3–4, as this effect is well established in other settings [31, 32]. We considered an increase of 2.38 ml/min per kg (i.e. +10.9%) in VO_2_peak as clinically relevant based on a minimal clinically important difference of 2 ml/min per kg or 6% reported in studies including patients with other chronic diseases [33–35]. Also the accompanying improvements in global and physical aspects of Quality of Life (four included studies assessing Quality of Life found clinical relevant changes) indicated the improvement in VO_2_peak being of clinical relevance [21, 25–27]. Aerobic training has been shown to effect several central and peripheral CV adaptations such as improved cardiac output, decreased peripheral vascular resistance, higher blood volume, expanded capillary volume and increased peripheral O_2_-extraction, improving maximal oxygen uptake [36], with shear stress as key factor in vascular adaptations [37]. It is well documented that plasma volume expands up to 25% after 10–14 exercise sessions through increased plasma albumin levels and sodium retention in sedentary healthy subjects. A sustained 8–10% higher plasma compared to erythrocyte volume has also been noted after 30 days of training in sedentary people [38].

Sympathetic vasomotor over-activity is already present in the early stages of CKD and might play a role in the further deterioration of kidney function [39, 40]. Aerobic exercise restores surrogate parameters of sympathetic nervous system over-activity in patients with CKD stages 3–4, possibly through stimulation of vascular and renal baroregulatory systems on expanded blood volume [19, 41, 42]. Exercise training is a potential stimulus of vasodilation of the efferent renal arteriole in CKD, suggesting a downregulation of the renin-angiotensin system [43]. Indeed, Cornelissen and Fagard reported a 20% lower plasma renin activity due to aerobic exercise training in healthy sedentary normo- and hypertensive subjects [44].

Endothelial dysfunction is already present in early stages of CKD. The efferent renal arteriole demonstrates a constriction in response to glomerular endothelial injury [45] and may eventually contribute to further deterioration in eGFR and progression to renal microvascular disease [46]. A late-onset decrease in oxidative stress and increase in anti-oxidative factors due to exercise training could prevent progressive fibrosis in the kidneys [47, 48]. Exercise-induced blood volume expansion likely contributes to increased renal blood flow and shear stress in the kidney at rest and during exercise [49–51], the latter stimulating improvements in endothelial function [52]. However, Van Craenenbroeck et al [26] and Headley et al [27] reported no effect on flow-mediated dilation in patients with CKD stages 3–4 after a 12 and 16 weeks aerobic training regimen respectively. As a possible explanation, the authors advance the relatively short intervention period, which might not be enough to reverse established endothelial dysfunction. The results of this meta-analysis do not confirm a beneficial effect of aerobic exercise training on eGFR as a proxy for kidney function (Fig 4). However, they support the hypothesis that exercise might be beneficial in slowing down the progression CKD (Fig 3). Hence, as a preventive strategy, aerobic exercise training might be useful in patients with CKD stages 3–4.

Blood pressure is recognized as a critical remediable aspect in the maintenance of kidney function [37]. Hypertensive subjects experience a more substantial decrease in blood pressure by exercise training than normotensive subjects; underlying mechanisms to this training effect in (non-)healthy hypertensive subjects are improved vascular function (vascular remodelling and endothelial function), decreased sympathetic vasomotor over-activity and downregulation of the renin-angiotensin system [44]. However, the current meta-analysis did not show significant alterations in blood pressure by aerobic exercise training [53]. Our results are in line with the Cochrane review which reported no change in blood pressure following CV exercise. The Cochrane review, however, found a decrease in systolic and diastolic blood pressure in studies describing high intensity aerobic training (>60% VO_2_peak) and in studies combining aerobic and resistance training [11]. This meta-analysis included one study with such intensities [20] and four using combined exercise training [1, 19, 21, 24], and sensitivity analysis excluding these citations did not alter results. Due to a generally well-controlled blood pressure (<140/90 mmHg) at baseline [54], a great effect-size in blood pressure was not expected. Other possible explanations for the lack of significant change in blood pressure could be the use of and change in antihypertensives masking training effects (i.e. only four studies described unchanged medical therapy [22, 23, 25, 26]) and analysis being of low power since blood pressure was examined as secondary outcome in included studies. An unexpected decrease in systolic blood pressure by standard care was observed.

A moderate and high degree of heterogeneity (I^2^ = 50% and I^2^ = 90%) was found in the meta-analysis of eGFR and VO_2_peak respectively. Part of the heterogeneity in primary outcome might be due to age as elderly experience a smaller training effect size compared to younger subjects [55, 56]. The three studies covering the oldest participants noted a lower effect size on eGFR [1, 20, 21]. Analyses excluding these studies resulted in an improved eGFR of 3.43 ml/min per 1.73m^2^ [1.62; 5.23] with a negligible observed heterogeneity (I^2^<25%). Also exclusion of the three studies with the highest mean BMI values [1, 19, 20] resulted in a negligible heterogeneity and subgroup analyses showed favouring results for patients with better body weight control. A detrimental correlation between BMI and oxidative stress in patients with CKD stages 3–4 [57] could result in additional impairments of endothelial function ultimately obliterating exercise training effects on eGFR. It must be noted that two of three studies with mean BMI ≥32 kg/m^2^ are among those covering the oldest participants [1, 20]. Heterogeneity can partially if not almost fully be explained by between-study differences in age and BMI. Even though training modalities are determinants of treatment effects sizes for CV outcomes in patients with CKD [52], nor analyses on training type and duration nor differences in supervision, frequency or intensity of training could explain the high heterogeneity. However, exclusion of the four studies using both aerobic and resistance exercise training also resulted in a negligible heterogeneity (I^2^ = 19%). Sensitivity analysis excluding studies which also included patients with CKD stage 2 showed no alterations in effect size and heterogeneity (i.e. 2.16 ml/min per 1.73m^2^; I^2^ = 50% in the current analysis vs. 2.98 ml/min per 1.73m^2^; I^2^ = 56% in the sensitivity analysis for eGFR, p = 0.08 for subgroup analysis) [19–21].

It should be noted that muscle gain following physical exercise also affects eGFR measurements as it increases serum creatinine levels, thereby decreasing eGFR based on equations using creatinine. Hence, an exercise-induced increase in muscle mass may underestimate beneficial effects on kidney function as absolute improvements in eGFR may have been more pronounced than observed.

Quality and strength of evidence regarding VO_2_peak and blood pressure are low to very low, mainly due to high risk of bias and heterogeneity. However, exclusion of articles scoring high risk of bias on at least two different criteria did not alter the overall effect. The pooled findings of change in eGFR following aerobic exercise were rated as of moderate strength.

Even though this article could have different conclusions than the Cochrane review, in particular because only RCTs including patients with CKD stages 3–4 were selected, similar findings were retrieved for blood pressure and VO_2_peak [11].

### Limitations

This review has some contextual limitations and reasonable risk of bias. Selective reporting bias could result from retrospective registration of our protocol. Sedentary patients are less likely to engage in exercise intervention trials, for this reason, selection bias can be a pitfall in our meta-analysis, and results do thus not necessarily apply to the overall population with CKD stages 3–4. No studies were included reporting measured GFR and two estimation equations for eGFR were used with a rather low correlation to measured methods (i.e. r = 0.41 for the CKD-EPI and r = 0.27 for the MDRD) and to each other, inducing heterogeneity and additional bias [58]. Hydration status and muscle mass are undisputable confounders in the effects of aerobic exercise training on eGFR and should be taken into account in future studies. Outcome analyses were at risk of insufficient statistical power of individual studies since not more than 3 included studies described a similar primary outcome. Heterogeneity could be overestimated in our meta-analysis including only a small number of studies as we used the DerSimonian-Laird method. However, this is the standard approach applied by Review Manager 5.3.5 [59].

## Conclusions and guidelines for further research

This meta-analysis supports a beneficial effect of aerobic exercise on glomerular filtration rate and exercise tolerance in patients with CKD stages 3–4 when compared to standard care. Given the heterogeneous data, the lack of studies on hard and patient relevant endpoints, and at best low to moderate methodological quality of studies, this meta-analysis should act as an impetus for larger and more conclusive interventional research of high methodological quality (e.g. by examining measured GFR as primary outcome and with hydration status as covariate). Consensus regarding exercise prescription for this vulnerable population can only be reached when the question about which frequency, intensity, type and time of exercise is of most benefit, is answered.

## Supporting information

S1 Checklist PRISMA checklist. (DOC)

S1 Table Search strategy. (DOCX)

S1 Fig Funnel plot between-groups analysis for estimated glomerular filtration rate. (TIF)

S2 Fig Funnel plot between-groups analysis for systolic blood pressure. (TIF)

S3 Fig Funnel plot between-groups analysis for diastolic blood pressure. (TIF)

S4 Fig Funnel plot between-groups analysis for VO_2_peak. (TIF)

S5 Fig Funnel plot between-groups analysis for body mass index. (TIF)

S6 Fig Forest plots within-group analysis (control group). (1) glomerular filtration rate (ml/min per 1.73m^2^), (2) systolic blood pressure (mmHg), (3) diastolic blood pressure (mmHg) and (4) VO_2_peak (ml/kg/min)
(TIF)

S7 Fig Forest plot between-groups analysis of body mass index (kg/m^2^). (TIF)

S8 Fig Subgroup analysis of eGFR in studies with mean age <60 vs. ≥60 years old. (TIF)

S9 Fig Subgroup analysis of eGFR in studies with mean body mass index <32 vs. ≥32 kg/m^2^. (TIF)

S10 Fig Subgroup analysis of eGFR in studies with intervention duration ≥26 vs. <26 weeks. (TIF)

S11 Fig Subgroup analysis of eGFR in aerobic exercise training vs. the combination of aerobic and resistance exercise training. (TIF)
