# Supplementary material for: The effects of aerobic exercise on eGFR, blood pressure and VO2peak in patients with chronic kidney disease stages 3-4: A systematic review and meta-analysis
Source: PLoS One. 2018 Sep 11;13(9):e0203662. doi: 10.1371/journal.pone.0203662 (PMC6133282; doi:10.1371/journal.pone.0203662)
Supplement: S1 Table — (DOCX) [file pone.0203662.s002.docx]

S2 Table: **search strategy**

|  | PubMed | n | Web of Science | n | Embase | n |
| --- | --- | --- | --- | --- | --- | --- |
| P  +  I  +  O | ("Renal Insufficiency, Chronic"[Mesh] OR “Chronic Kidney Disease” OR “Chronic Renal Failure” OR “Pre-dialysis”)  **AND**  ("Exercise"[Mesh] OR "Circuit-Based Exercise"[Mesh] OR "Motor Activity”[Mesh] OR "Rehabilitation"[Mesh] OR “aerobic exercise training” OR “aerobic exercise” OR “exercise training”)  **AND**  ((“Glomerular Filtration Rate"[Mesh] OR “GFR” OR “Kidney Function” OR “renal function” OR "Blood Urea Nitrogen"[Mesh] OR "Radioisotope Renography”[Mesh]) OR ("Heart Rate”[Mesh]) OR (“Blood Pressure“[Mesh]) OR (“oxygen consumption”) OR (“VO2peak”) OR (“aerobic capacity”)) | 417 | TS = (“Chronic Renal Insufficiency” OR “Chronic Kidney Disease” OR “Renal Insufficiency” OR “Kidney Insufficiency” OR “Renal Failure”)  **AND**  TS = (“Exercise" OR “Aerobic Exercise” OR “Aerobic Training” OR “Physical Exercise” OR “Physical Training” OR “Rehabilitation”)  **AND**  TS = (“Glomerular Filtration Rate” OR "Blood Urea Nitrogen” OR "Radioisotope Renography” OR “Kidney Disease Trajectory” OR “Kidney Function” OR “Renal Function” OR "Heart Rate” OR “Blood Pressure“ OR “Endothelial Function” OR “Systolic Pressure” OR “Diastolic Pressure” OR “Oxygen Consumption” OR “VO_2_peak” OR “Aerobic Capacity” OR “Exercise Capacity”) | 875 | ('chronic kidney failure'/exp OR 'chronic kidney disease' OR 'chronic kidney disorder' OR 'chronic kidney failure' OR 'chronic kidney insufficiency' OR 'chronic nephropathy' OR 'chronic renal disease' OR 'chronic renal failure' OR 'chronic renal insufficiency' OR 'kidney chronic failure' OR 'kidney disease, chronic' OR 'kidney failure, chronic' OR 'kidney function, chronic disease' OR 'renal insufficiency, chronic')  **AND**  ('aerobic exercise'/exp OR 'aerobic exercise' OR 'aerobics exercise' OR 'exercise, aerobic' OR 'exercise'/exp OR 'exercise' OR 'exercise capacity' OR 'exercise performance' OR 'exercise training' OR 'physical exercise')  **AND**  ('glomerulus filtration rate'/exp OR 'gfr (glomerulus filtration rate)' OR 'glomerular filtration rate' OR 'glomerulofiltration rate' OR 'glomerulus filtration rate' OR 'kidney gfr' OR 'kidney glomerulus filtration rate' OR 'endothelial function'/exp OR 'flow mediated dilation'/exp OR 'kidney function'/exp OR 'function, kidney' OR 'kidney function' OR 'kidney physiology' OR 'renal function' OR 'heart rate'/exp OR 'cardiac frequency' OR 'cardiac rate' OR 'heart frequency' OR 'heart rate' OR 'rate, heart' OR 'ventricle rate' OR 'blood pressure'/exp OR 'blood pressure' OR 'blood tension' OR ‘VO2peak’ OR ‘exercise capacity’ OR ‘VO2max’ OR ‘maximal oxygen uptake’ OR ‘exercise performance’) | 1038 |
| Databases were searched until February 2018  No additional filters were applied  No publication date was restricted | | | | | | |
